# Uncovering key biopsychosocial risk factors in non-violent suicide attempts: evidence from a Hungarian cross-sectional study

**DOI:** 10.3389/fpsyt.2025.1626808

**Published:** 2025-08-20

**Authors:** Noemi Monika Szeifert, Barnabás Oláh, Barbara Sebok, Xenia Gonda

**Affiliations:** ^1^ Doctoral School of Psychology, ELTE Eötvös Lóránd University, Budapest, Hungary; ^2^ Department of Sports Medicine, Semmelweis University, Budapest, Hungary; ^3^ Department of Behavioural Sciences, Faculty of Medicine, University of Debrecen, Debrecen, Hungary; ^4^ Clinical Psychology Center of CC, Health Care Service Units, University of Debrecen Clinical Centre, Debrecen, Hungary; ^5^ Semmelweis University School of PhD Studies Workgroup for Science Management, Dr. Manninger Jenő Trauma Center, Budapest, Hungary; ^6^ Department of Clinical Psychology, Semmelweis University, Budapest, Hungary; ^7^ Department of Psychiatry and Psychotherapy, Semmelweis University, Budapest, Hungary; ^8^ NAP3.0-SE Neuropsychopharmacology Research Group, Hungarian Brain Research Program, Semmelweis University, Budapest, Hungary

**Keywords:** suicide risk, psychiatric disorders, gender differences, single and multiple attempters, suicide prevention

## Abstract

**Introduction:**

This Hungarian cross-sectional study examined patterns and differences in suicide risk factors across various suicidality groups, including individuals with single or multiple suicide attempts, as well as gender-specific variations. Additionally, it explored these risk factors within a biopsychosocial framework to offer a comprehensive understanding of their interconnected effects.

**Materials and methods:**

A total of 300 psychiatric inpatients were recruited from Péterfy Sandor Hospital in Budapest, Hungary, including 146 individuals (48.67%) with a history of suicide attempts and 154 (51.33%) without such a history. Participants ranged in age from 18 to 85 years, with a mean age of 37.98 years (SD = 12.80 for suicide attempters, 13.72 for non-attempters). The overall sample comprised 83 males (27.7%) and 217 females (72.3%). Logistic regression analysis was conducted to assess the influence of demographic characteristics, life history variables, and psychiatric diagnoses on suicide risk, aiming to identify significant predictors of suicide attempts within a biopsychosocial framework.

**Results:**

Depression was the most prevalent psychiatric diagnosis in the sample. Significant predictors of suicide attempts included family history of suicide (OR = 2.283, *p* = 0.015), prescription drug misuse (OR = 1.900, *p* = 0.047), and nicotine dependence (OR = 1.869, *p* = 0.035). In repeated suicide attempts, bipolar disorder (OR = 5.761, *p* = 0.006), borderline personality disorder (OR = 5.132, *p* = 0.003), depression (OR = 4.064, *p* = 0.004), and job loss (OR = 4.348, *p* = 0.031) emerged as the strongest predictors. Among men, job loss (OR = 4.074, *p* = 0.014) was a prominent risk factor, while among women, having two or more children (OR = 2.740, *p* = 0.036) and a family history of suicide (OR = 2.459, *p* = 0.028) significantly increased suicide risk. Relationship conflict was also associated with higher risk in women (OR = 0.382, *p* = 0.035).

**Conclusions:**

Our research supports the notion that suicide risk factors interact with one another, and in certain cases, their effects may be synergistic—mutually reinforcing—rather than antagonistic. Similarly, protective factors also appear to amplify each other’s impact, suggesting a cumulative and interactive model of both risk and resilience.

## Introduction

Suicide represents a significant global public health issue, ranking among the leading causes of death worldwide. According to data from the World Health Organization (WHO), over 700,000 individuals die by suicide each year, equivalent to one death every 40 seconds. Suicide is a phenomenon that affects people across all age groups and occurs throughout the lifespan (1, 2).

In Hungary, suicide has historically been a significant public health concern, with the country having had one of the highest suicide rates in the world at one point in time (3, 4). However, over the past few decades, there has been a notable decline in suicide rates. This reduction is attributed to several factors, including improved mental health services, greater access to psychiatric care, and increased public awareness surrounding mental health issues. The introduction of antidepressants, particularly SSRIs, in the 1990s, also played a role in reducing suicides. Additionally, economic improvements and social support systems have also contributed to this downward trend 5–7).

Despite these improvements, in 2023, a total of 1,593 (8) individuals died by suicide in Hungary, a rate that remains above the European average. Hungarian studies have consistently identified depression, bipolar disorder, anxiety disorders, and substance use—especially smoking and alcohol—as key risk factors for both suicide attempts and completed suicides (3, 9–15). A disadvantaged background—characterized by low socio-economic status, a history of parental suicide attempts, and exposure to psychological adversities—has also been consistently associated with an elevated risk of suicide, particularly in the presence of an untreated psychiatric disorder (16–18). These patterns are notably prevalent among middle-aged and older men, and higher suicide rates are concentrated in rural areas and the southeastern region (Rihmer et al., 2013). Continuous efforts are needed to sustain progress by improving early detection, reducing stigma, and expanding access to care (19).

Findings suggest that the COVID-19 pandemic’s socio-economic and psychological impacts may have exacerbated factors contributing to suicide in Hungary. The data underscores the importance of targeted mental health interventions and support systems, especially for the most affected demographics and regions (20, 21).

### Suicide and methods

A suicide attempt is defined as a self-initiated, potentially harmful behavior undertaken with the intent to end one’s life, which does not result in death. It is influenced by a complex interplay of psychological, environmental, and biological factors (22). In suicide research, methods are often categorized as *violent* or *non-violent* based on the nature and lethality of the act (23). This classification aids in understanding the characteristics and risk factors associated with different suicide methods. *Violent* methods typically involve direct physical force and are associated with a high risk of immediate lethality, such as firearms/gunshots, hanging or strangulation, and blunt force injuries (e.g., jumping from a high place or stepping in front of a train). *Non-violent* suicide attempts typically refer to methods that do not involve physical trauma, such as overdosing on medication, poisoning, or asphyxiation (17). The risk factors for non-violent suicide attempts can overlap with general suicide risk factors, but may also have unique aspects related to method choice.

The most common and robust predictors of suicide attempts, in hierarchical order, are (24): *Psychiatric* and *psychological* disorders play a significant role in influencing suicide methods, particularly depression, bipolar disorder, and anxiety disorders (9, 11, 25). These are among the most common underlying mental health conditions associated with non-violent and violent suicide attempts, along with other serious mental illnesses (25). Personality disorders, particularly borderline personality disorder, are also strongly linked to suicidal behavior due to their association with impulsivity and self-harm (26, 27). A history of previous suicide attempts, especially non-violent ones, increases the risk of future attempts (17, 28). Substance abuse, including alcohol and drug misuse, further elevates suicide risk by impairing judgment and increasing impulsivity (11, 29, 30). Additionally, hopelessness and feelings of worthlessness are powerful psychological predictors of suicidal behavior (31). *Medical* and *physical health factors* are important contributors to suicide risk, particularly concerning method choice. Chronic illnesses such as cancer, chronic pain syndromes, and other debilitating diseases are strongly associated with increased suicidal ideation and behavior, often due to feelings of hopelessness, loss of autonomy, and perceived burden on others (32, 33). Individuals living with chronic medical conditions frequently have greater access to prescription medications, which may facilitate non-violent suicide attempts such as overdose (34, 35). The availability and familiarity with medications used for pain or symptom management can inadvertently increase the risk, especially in the context of psychological distress or comorbid mental health conditions (36, 37). *Demographic factors* influencing suicide methods include gender, age, and socioeconomic status. Women are more likely to attempt suicide using non-violent methods such as poisoning or overdose, while men tend to use more violent means (4, 38). Adolescents, young adults, and older adults may also favor non-violent methods (39). Additionally, individuals with higher education and socioeconomic status have sometimes been linked to non-violent methods, possibly due to better access to prescription medications (40). *Social* and *environmental factors* significantly influence suicide risk, particularly in shaping an individual’s vulnerability during times of crisis. Isolation and lack of social support have been consistently associated with increased suicidal ideation, as loneliness and the absence of close interpersonal relationships can exacerbate feelings of despair and hopelessness (41, 42). Recent life stressors, including relationship breakups, job loss, financial hardship, or involvement with the legal system, are also known to trigger suicidal behavior, especially in individuals with limited coping resources (3, 41, 43). A history of abuse or trauma, such as childhood maltreatment, sexual abuse, or exposure to violence, further compounds the risk by disrupting emotional regulation and trust in others (44–46). Cultural factors, including mental health stigma, can discourage individuals from seeking professional help or disclosing distress, a phenomenon particularly prevalent among minority populations such as the Hungarian Roma, where cultural marginalization and institutional distrust may amplify vulnerability (47, 48). *Cognitive* and *behavioral factors* play a crucial role in suicide risk, including the likelihood and method of attempts. Impulsivity is a well-established risk factor, even in cases where non-violent suicide attempts are planned, as impulsive individuals may act quickly in response to distress without fully considering the consequences (17, 49). Low problem-solving skills, particularly in managing interpersonal stressors or navigating complex emotional situations, have also been linked to an increased risk of suicidal behavior, as individuals may perceive suicide as the only escape from unsolvable problems (50). Additionally, the use of poor or maladaptive coping mechanisms—such as avoidance, denial, or substance use—can further exacerbate emotional pain and reduce resilience, increasing the likelihood of suicide attempts (51). *Family* and *genetic factors* are critical in understanding suicide risk, as both inherited traits and environmental influences can contribute. A family history of suicide or mental illness is associated with a higher likelihood of suicidal behavior, suggesting both genetic predispositions and the influence of modeled or learned behaviors within the family context (52). Additionally, family conflict and dysfunction—such as high levels of criticism, lack of emotional support, or exposure to domestic violence—can increase psychological distress and impair emotional regulation, further elevating suicide risk, particularly among adolescents and young adults (53). These factors often interact, compounding vulnerability in individuals already at risk due to mental health or cognitive challenges. *The availability of means* is a critical determinant of suicide risk, as access to lethal methods significantly increases the likelihood of both suicide attempts and fatalities. Easy access to medications or toxic substances, such as prescription drugs or household chemicals, raises the risk of impulsive or planned non-violent suicide attempts, particularly in individuals experiencing acute psychological distress (54). When such means are not securely stored or adequately restricted, such as unsecured medications in the home, the probability of use in a suicide attempt increases substantially (55). Restricting access to lethal means is one of the most effective strategies in suicide prevention, underscoring the importance of environmental safety measures. While numerous risk factors increase vulnerability to suicide, *protective factors* can buffer against these risks by enhancing resilience and reducing the likelihood of suicidal behavior. Strong social support networks serve as a critical protective element, offering emotional connection and reducing feelings of isolation (56). Access to mental health care, including early intervention and ongoing treatment, has been shown to significantly decrease suicide risk, especially when combined with effective therapeutic approaches (57, 58). Individuals with effective coping and problem-solving skills are better equipped to manage stress and emotional crises, reducing reliance on maladaptive behaviors (59). Cultural or religious beliefs that discourage suicide may provide a moral or existential framework that deters self-harm (60). Additionally, engagement in meaningful activities—such as work, regular physical activity, hobbies, or volunteering—can foster a sense of purpose and belonging, which is associated with lower suicide risk (61). These protective factors serve as crucial counterbalances to the cognitive, psychological, and social vulnerabilities associated with suicide.

Our research aims to analyze the main non-violent suicide risk factors in an inpatient psychiatric sample within a biopsychosocial framework, with a focus on identifying the most significant factors and their interactions.

## Methods

### Sample

Participants in the patient group were recruited from the Crisis Intervention and Psychiatric Ward at Péterfy Sándor Hospital in Budapest, Hungary, between January 1, 2016, and December 31, 2021. The sample was derived from a comprehensive research project utilizing paper-and-pencil questionnaires, which inherently limited the sample size. Although approximately 4,000 to 5,000 patients were admitted to the referenced department at Péterfy Hospital during the study period, the use of detailed self-report instruments restricted the number of participants who could be feasibly included. Psychiatric diagnoses, along with assessments of suicide risk and underlying motives, were conducted using the Structured Clinical Interview for DSM-IV (SCID-IV), administered by psychiatrists and trained clinical psychologists. Diagnoses were further supported by classifications based on the International Classification of Diseases, 10th Revision (ICD-10). Diagnoses were recorded at the time of admission to the psychiatric ward. Individuals were excluded from the comprehensive study if they were experiencing an acute psychotic episode, current alcohol or substance abuse, schizophrenia, a history of traumatic brain injury or neurological disorders, severe medical conditions, or had engaged in parasuicidal behavior. Participation in the study was entirely voluntary, and all participants provided written informed consent for the extraction of relevant data from the hospital’s electronic medical record system. The study received ethical approval from the Institutional Review Board of Péterfy Sándor a Hospital and Outpatient Center (Approval No. 25/2016). The authors affirm that all procedures contributing to this research comply with the ethical standards of the relevant national and institutional committees on human experimentation, as well as with the Helsinki Declaration of 1975, as revised in 2013.

### Measures

Data were collected through structured clinical interviews conducted by psychiatrists and trained clinicians, supplemented by information retrieved from the hospital’s central electronic medical record system. The analysis included a range of variables: gender, age, place of residence, educational level, marital status, number of children, employment status, financial status, psychiatric diagnosis, and various forms of substance dependence, including alcohol, medication, other substances, and nicotine. Additional data were collected on suicide motives and family history of suicide. Participants also completed a self-report questionnaire addressing whether they were born from an unintended pregnancy, whether their mother had attempted an induced abortion or suicide during pregnancy (coding was as follows: 1 = Yes, 2 = No, 3 = No information available), and whether they had undergone an induced abortion. Participants who had experienced the loss of a child were also asked to complete relevant sections of the questionnaire.

### Statistical analysis

Statistical analyses were conducted using IBM SPSS Statistics, version 23. Initially, the demographic characteristics of the sample were described using means and standard deviations for continuous variables, and proportions for categorical variables, stratified by history of suicide attempts. To assess bivariate associations between suicide risk factors and history of suicide attempts, Pearson’s chi-square tests were employed. Comorbidity of psychiatric diagnoses among individuals with a history of suicide attempts was examined using Phi coefficients (φ).

Subsequently, logistic regression models were constructed to investigate the associations between various risk factors and suicidal behavior, while adjusting for demographic variables, including gender, age, place of residence, educational attainment, relationship status, number of children, employment status, and subjective financial situation. To enhance the interpretability and stability of the models, certain variables were recategorized as follows: place of residence (city, town, or village); education (primary, secondary, or tertiary); relationship status (single *vs*. in a relationship); and financial status (below average, average, or above average). Finally, *post hoc* analyses were performed using the adjusted Wald test.

## Results

### Descriptive statistics

Among the 300 participants, 146 individuals (48.67%) reported a history of suicide attempts, while 154 (51.33%) had no such history. Participant ages ranged from 18 to 85 years, with a mean age of 37.98 years (SD = 12.80 for those with a history of suicide attempts, and SD = 13.72 for those without). Of the total sample, 83 individuals (27.7%) were male and 217 (72.3%) were female (**Table 1**). Among those with a history of suicide attempts, 40 (27.5%) were male and 106 (72.6%) were female, reflecting the well-documented pattern that non-violent suicide attempts and psychiatric service utilization are more prevalent among women. This finding is consistent with existing literature indicating that females are generally more likely than males to seek psychological help.

**Table 1:** Demographic characteristics of the samples.

	Suicidal behavior (n=146)	No suicidal behavior (n=154)	Test statistics^i^
Gender n (%)			
Male	40 (27.5%)	43 (27.9%)	χ²(1) = 0.01, p = 0.92
Female	106 (72.6%)	111 (72.1%)	
**Age mean (SD)**	37.25 (12.80)	38.68 (13.72)	F(298) = 2.81, p = 0.095
Residence n (%)			
Capital city	82 (56.2%)	88 (57.1%)	χ²(3) = 2.477, p = 0.480
Regional city	13 (8.9%)	17 (11.0%)	
Rural town	32 (21.9%)	37 (24.0%)	
Village	19 (13.0%)	12 (7.8%)	
Educational level n (%)			
Primary	13 (8.9%)	13 (8.4%)	χ²(3) = 3.547, p =0.315
Vocational	31 (21.2%)	25 (16.2%)	
High school	63 (43.2%)	60 (39.0%)	
Higher education	39 (26.7%)	56 (36.4%)	
Marital status n (%)			
Single	63 (43.2%)	53 (34.4%)	χ²(4) = 5.886, p = 0.208
In relationship	26 (17.8%)	34 (22.1%)	
Married/cohabiting	42 (28.8%)	48 (31.2%)	
Divorced	13 (8.9%)	11 (7.1%)	
Widowed	2 (1.4%)	8 (5.2%)	
Subjective financial status n (%)			
Very Poor	9 (6.2%)	12 (7.8%)	χ²(4) = 3.310, p = 0.507
Poor	29 (19.9%)	19 (12.3%)	
Moderate	76 (52.1%)	87 (56.5%)	
Good	28 (19.2%)	32 (20.8%)	
Excellent	4 (2.7%)	4 (2.6%)	
Number of children n (%)			
None	78 (53.4%)	87 (56.5%)	χ²(2) = 0.515, p = 0.773
1	25 (17.1%)	22 (14.3%)	
2 or more	43 (29.5%)	45 (29.2%)	
Employment status n (%)			
Full time employee	90 (61.6%)	84 (54.5%)	χ²(2) = 1.888, p = 0.389
Unemployed	18 (12.3%)	26 (16.9%)	
Other (part time, retired, student)	38 (26.0%)	44 (28.6%)	

i Indicates the application of Pearson’s chi-squared test or Pearson’s T-test.

Concerning the frequency of suicide attempts among attempters, 56.8% reported a single attempt, 21.2% reported two attempts, and 22.0% reported three or more. The age at first suicide attempt varied considerably: 11.0% of participants had their first attempt before the age of 18, 33.6% between the ages of 18 and 29, 28.1% between 30 and 44, 16.4% between 45 and 59, and 4.1% at age 60 or older. Post-discharge follow-up data indicated that 24.7% of individuals with a prior suicide attempt made a subsequent attempt within one month, 11.0% within three months, 5.5% within six months, and 12.3% within one year of discharge.

### Risk factors

Concerning familial suicide risk factors, a family history of suicide was significantly more common among individuals with a history of suicide attempts (43.8%) than among those without such a history (22.7%) (χ²(1) = 15.103, p <.001). Conversely, being born as a result of an unintended pregnancy was more frequently reported among suicide attempters (40.0%) compared to non-attempters (29.0%), although this difference did not reach statistical significance. Similarly, 10.5% of individuals with a history of suicide attempts and 7.2% of non-attempters reported that their mother had either attempted abortion or engaged in suicidal behavior during the pregnancy. The experience of losing a child was reported by 8.3% of attempters and 6.6% of non-attempters, while a personal history of abortion was noted in 24.2% and 21.3% of individuals in the respective groups (**Table 2**).

**Table 2:** Descriptive statistics of familial suicidal risk factors and psychiatric disorders in the samples and the motives of the latest suicide attempt.

	Attempted suicide (n=146)	No suicidal behavior (n=154)	Test statistics^i^
Familial suicidal risk factors			
Family history of suicide	64 (43.8%)	35 (22.7%)	χ²(1) = 15.103, p<0.001
Born from unintended pregnancy	50 (40.0%)	37 (29%)	χ²(1) = 3.134, p = 0.077
Maternal abortion or suicide attempt during pregnancy	13 (10.5%)	9 (7.2%)	χ²(1) = 0.833, p = 0.361
Child bereavement	10 (8.3%)	9 (6.6%)	χ²(1) = 0.254, p = 0.615
Personal history of abortion	29 (24.2%)	29 (21.3%)	χ²(1) = 0.294, p = 0.588
Mood, anxiety, personality disorders			
Depression disorder	73 (50.0%)	67 (43.5%)	χ²(1) = 1.27, p = 0.260
Bipolar disorder	26 (17.8%)	24 (15.6%)	χ²(1) = 0.267, p = 0.605
Anxiety disorder	71 (48.6%)	88 (57.1%)	χ²(1) = 2.180, p = 0.140
Emotionally unstable personality disorder	49 (33.6%)	40 (26.0%)	χ²(1) = 2.068, p = 0.150
Personality disorder not other specified	31 (21.2%)	48 (31.2%)	χ²(1) = 3.814, p = 0.051
Substance abuse disorders			
Alcohol dependence	52 (35.6%)	39 (25.3%)	χ²(1) = 3.757, p = 0.053
Illicit drug dependence	23 (15.8%)	22 (14.3%)	χ²(1) = 0.127, p = 0.722
Prescription drug misuse	46 (31.5%)	26 (16.9%)	χ²(1) = 8.787, p = 0.003
Nicotine dependence	61 (41.8%)	45 (29.2%)	χ²(1) = 5.175, p = 0.023
Motives			
Financial hardship	23 (15.8%)	–	–
Job loss	19 (13.3%)	–	–
Relationship conflict	54 (37.0%)	–	–
Divorce/Breakup	46 (31.5%)	–	–
Loss of close relative	13 (8.9%)	–	–
Chronic illness	25 (17.1%)	–	–
Other	54 (37.0%)	–	–

i Indicates the application of Pearson’s chi-squared test.

The distribution of psychiatric diagnoses within the total sample of 300 psychiatric patients was as follows: major depressive disorders were diagnosed in 140 individuals (46.7%), bipolar disorder in 50 (16.7%), and anxiety disorders in 159 participants (53.0%). Emotionally unstable personality disorder (emotionally unstable type) was identified in 89 individuals (29.7%), while 79 participants (26.3%) met criteria for unspecified personality disorder. Substance use disorders were also prevalent: 91 individuals (30.3%) were diagnosed with alcohol use disorder, 45 (15.0%) with illicit drug use disorder, and 72 (24.0%) with prescription drug misuse. Nicotine dependence was reported by 106 individuals (35.3%). A detailed overview of psychiatric diagnoses is provided in **Table 2**.

Among those with a history of suicide attempts, the most frequently diagnosed psychiatric disorders included major depressive disorders (73 individuals, 50.0%), anxiety disorders (71 individuals, 48.6%), emotionally unstable personality disorder (49 individuals, 33.6%), bipolar disorder (26 individuals, 17.8%), and unspecified personality disorder (31 individuals, 21.2%). In terms of substance use, the most commonly reported conditions among suicide attempters were nicotine dependence (61 individuals, 41.8%), followed by alcohol use disorder (52 individuals, 35.6%), prescription drug misuse (46 individuals, 31.5%), and illicit drug use disorder (23 individuals, 15.8%).

Regarding the reported motivations for the most recent suicide attempt (data available only for the attempter group), the most frequently cited reasons included relationship conflict (37.0%), divorce or breakup (31.5%), and financial hardship (15.8%). Additional motives were chronic illness (17.1%), job loss (13.3%), the loss of a close relative (8.9%), and other unspecified reasons (37.0%) (**Table 2**).

### Intercorrelations of psychiatric diagnoses among suicide attempters

**Table 3** presents the correlation matrix of psychiatric diagnoses among individuals with a history of suicide attempts, utilizing Phi coefficients (φ) to quantify the strength of associations between diagnostic categories. Consistent with clinical conventions, no co-occurrence of unipolar depression and bipolar disorder was observed in the sample, reflecting their generally mutually exclusive diagnostic status. Noteworthy findings include a significant negative association between depression and emotionally unstable personality disorder (φ = -0.189, p < 0.05), which may reflect diagnostic practices wherein clinicians preferentially assign one diagnosis over the other. Similarly, emotionally unstable personality disorder and unspecified personality disorder exhibited a significant negative correlation (φ = -0.298, p < 0.001), likely due to the clinical tendency to avoid assigning a nonspecific personality disorder diagnosis when a more specific diagnosis is established. Positive associations were identified between emotionally unstable personality disorder and illicit drug dependence (φ = 0.250, p < 0.05), as well as between alcohol dependence and both prescription drug misuse (φ = 0.388, p < 0.001) and nicotine dependence (φ = 0.298, p < 0.001). The strongest correlation within the matrix was observed between illicit drug dependence and nicotine dependence (φ = 0.396, p < 0.001), indicating a particularly robust comorbidity. These findings underscore the complex interplay between psychiatric disorders and substance use within this high-risk cohort.

**Table 3:** The correlation matrix of psychiatric diagnoses among suicide attempters.

	Depression disorder	Bipolar disorder	Anxiety disorder	Emotionally unstable pd.	Personality disorder nos.	Alcohol dependence	Illicit drug dependence	Prescription drug misuse	Nicotine dependence
Depression disorder	–	–	0.041	-0.189*	0.017	-0.143	-0.056	0.000	-0.014
Bipolar disorder	–	–	-0.131	0.010	-0.110	-0.10	-0.005	0.070	-0.031
Anxiety disorder	0.041	-0.131	–	-0.082	-0.103	-0.151	-0.045	-0.129	-0.037
Emotionally unstable pd.	-0.189*	0.010	-0.082	–	-0.298**	-0.017	0.250*	0.142	0.133
Personality disorder nos.	0.017	-0.110	-0.103	-0.298**	–	0.069	0.005	0.044	0.036
Alcohol dependence	-0.143	-0.10	-0.151	-0.017	0.069	–	0.189*	0.388**	0.298**
Illicit drug dependence	-0.056	-0.005	-0.045	0.250*	0.005	0.189*	–	0.192*	0.396**
Prescription drug misuse	0.000	0.070	-0.129	0.142	0.044	0.388**	0.192*	–	0.233*
Nicotine dependence	-0.014	-0.031	-0.037	0.133	0.036	0.298**	0.396**	0.233*	–

Phi coefficients (φ) indicate the strength of association, based on chi-square tests.

*p<0.05, **p<0.001.

### Demographic, familial and psychiatric predictors of suicidal behavior

Multivariate logistic regression models (**Table 4**) were employed to examine the associations between demographic characteristics, familial risk factors, mood, anxiety, and personality disorder diagnoses, as well as substance use, concerning suicidal behavior. Separate models were computed for each group of variables. Models 2, 3, and 4 were adjusted for demographic covariates.

**Table 4:** Modelling the associations of demographic variables, familial suicidal risk factors and psychiatric diagnoses with suicidal behavior.

	Suicidal behavior	
	OR^i^	p-value
Model 1: Demographics		
Gender (ref.: females)	0.971	0.913
Age (years)	0.977	0.083
Residence (ref.: city)		
Town	0.484	0.115
Village	0.552	0.155
Educational attainment (ref.: tertiary)		
Secondary	1.681	0.072
Primary	1.405	0.479
Relationship status: Single (ref.: in relationship)	1.414	0.183
Number of children (ref.: 2 or more)		
none	0.588	0.158
1	0.574	0.574
Employment status (ref.: full time)		
Unemployed	0.491	0.056
Other	0.697	0.697
Subjective financial status (ref.: above average)		
Average	0.771	0.418
Below average	1.257	0.558
Model 2: Familial suicidal risk factors		
Suicide in family history (ref.: no)	2.283	0.015
Was not a planned child (ref.: was planned)	1.460	0.305
Abortion was attempted at birth (ref.: no)	0.756	0.638
Loss of child (ref.: no)	1.943	0.321
Had an abortion (ref.: no)	0.937	0.864
Model 3: Mood, anxiety, personality disorders		
Depression disorder (ref.: no)	1.531	0.144
Bipolar disorder (ref.: no)	1.420	0.358
Anxiety disorder (ref.: no)	0.637	0.081
Emotionally unstable personality disorder (ref.: no)	1.245	0.513
Personality disorder nos. (ref.: no)	0.568	0.070
Model 4: Substance abuse disorders		
Alcohol dependence (ref.: no)	1.264	0.449
Illicit drug dependence (ref.: no)	0.517	0.110
Prescription drug misuse (ref.: no)	1.900	0.047
Nicotine dependence (ref.: no)	1.869	0.035

i Logistic regression models with entry method, all models were adjusted for gender, age, residency, education, relationship status, number of children, employment status and subjective financial status.

Among the demographic variables, no main effects reached statistical significance. However, a family history of suicide emerged as a significant predictor of suicidal behavior (OR = 2.283, p = 0.015), indicating more than a twofold increase in risk. Other familial factors were not significantly associated with suicidal behavior.

None of the mood, anxiety, or personality disorder diagnoses—including depression, bipolar disorder, anxiety disorders, emotionally unstable personality disorder, and unspecified personality disorders—significantly differentiated between individuals with and without suicidal behavior within the clinical sample. Nonetheless, non-significant trends suggesting increased risk were observed for depression, anxiety disorders, and emotionally unstable personality disorder.

In contrast, substance use variables were more predictive of suicidal behavior in this clinical population. Nicotine dependence was significantly associated with suicidal behavior (OR = 1.869, p = 0.035), corresponding to an approximately 87% increased risk. Prescription drug misuse also showed a significant association (OR = 1.900, p = 0.047), suggesting nearly double the risk. Other substance-related variables did not reach statistical significance in the sample.

### Gender differences

Gender-specific differences were observed in the predictors of suicidal behavior (**Table 5**). Among females, increasing age was significantly associated with a reduced likelihood of suicidal behavior (OR = 0.955, p = 0.007), corresponding to a 4.5% decrease in risk for each additional year of age. Furthermore, women with two or more children exhibited significantly higher odds of suicidal behavior compared to those without children (OR = 2.740, p = 0.036). A family history of suicide was also a significant predictor among women, associated with a 2.46-fold increase in risk (OR = 2.459, p = 0.028). In contrast, none of these variables demonstrated statistically significant associations with suicidal behavior among males.

**Table 5:** The differences of suicide predictors between gender groups.

	Suicidal behavior			
	Females		Males	
	OR^i^	p-value	OR^i^	p-value
Age (years)	0.955	0.007	1.011	0.656
Number of children (ref.: none)				
2 or more	2.74	0.036	0.856	0.826
Suicide history in family	2.459	0.028	1.496	0.672

i Logistic regression models using the entry method were adjusted for gender, age, place of residence, education level, relationship status, number of children, employment status, and subjective financial status.

Gender differences also emerged in the reported motives for suicidal behavior (**Table 6**). Men were significantly more likely to cite job loss as a contributing factor, with a 4.07-fold increased likelihood compared to women (OR = 4.074, p = 0.014). Conversely, relationship conflict was more frequently associated with suicidal behavior among women; men were 2.6 times less likely than women to report this as a motive (male [ref.: female]: OR = 0.382, p = 0.035).

**Table 6:** The associations of gender with suicide motives.

	Gender: male (ref.: female)	
	OR^i^	p-value
Job loss (ref.: no)	4.074	0.014
Relationship conflicts (ref.: no)	0.382	0.035

i Logistic regression models using the entry method were adjusted for gender, age, place of residence, education level, relationship status, number of children, employment status, and subjective financial status.

These findings underscore the importance of gender-sensitive approaches to suicide prevention. For women, interventions may be more effective when addressing familial dynamics and interpersonal stressors, whereas for men, economic stressors—particularly job loss—appear to constitute a more salient risk factor. A nuanced understanding of these gender-specific patterns is critical for the development of targeted and effective prevention strategies.

### Predictors of multiple suicide attempts

**Table 7** illustrates that among psychiatric diagnoses, depression emerged as a strong and significant predictor of repeated suicidal behavior, with affected individuals exhibiting more than a fourfold increase in the odds of multiple suicide attempts (OR = 4.064, *p* = 0.004). Bipolar disorder was similarly associated with a markedly elevated risk, conferring nearly a sixfold greater likelihood of multiple attempts compared to those without the diagnosis (OR = 5.761, *p* = 0.006). Likewise, emotionally unstable personality disorder was a robust predictor, linked to over a fivefold increase in the probability of recurrent suicide attempts (OR = 5.132, *p* = 0.003). These findings suggest that mood instability, affective dysregulation, and chronic emotional distress are critical factors in the recurrence of suicidal behavior.

**Table 7:** Modelling the predictors of multiple suicide attempts.

	Multiple suicide attempt (ref.: single)	
	OR^i^	p-value
Depressive disorder (ref.: no)	4.064	0.004
Bipolar disorder (ref.: no)	5.761	0.006
Emotionally unstable personality disorder (ref.: no)	5.132	0.003
Job loss (ref.: no)	4.348	0.031

i Logistic regression models employing the entry method were adjusted for gender, age, place of residence, education level, relationship status, number of children, employment status, and subjective financial status.

Moreover, job loss was identified as a significant psychosocial risk factor, increasing the odds of multiple suicide attempts by more than fourfold relative to individuals without this experience (OR = 4.348, *p* = 0.031).

Collectively, these results underscore the necessity of prioritizing specific psychiatric diagnoses—particularly depression, bipolar disorder, and emotionally unstable personality disorder—in the assessment of risk for repeated suicide attempts. They further highlight the importance of comprehensive, individualized evaluation and intervention strategies tailored to these high-risk profiles.

## Discussion

This study identified several key predictors of suicide attempts within a psychiatric inpatient population, encompassing a comprehensive range of demographic, psychiatric, and life history variables. Among all examined variables predicting suicide attempts, family history of suicide (OR = 2.283, p = 0.015) emerged as the most stable predictor. This finding supports previous research suggesting that genetic vulnerability and shared environmental stressors contribute to the intergenerational transmission of suicidal behavior (52; Rihmer et al., 2013). Consistent with a substantial body of literature, major depressive disorder also emerged as among the most robust and consistent predictors of suicidal behavior, particularly in cases involving repeated suicide attempts (OR=4.064, *p*=0.004). Notably, major depressive disorder diagnosis also demonstrated the highest prevalence within the sample (46.6%). This finding aligns with prior research indicating that major depressive disorder are among the most robust predictors of both suicidal ideation, intent and attempts (12, 25, 54, 62). Nicotine dependence was significantly associated with increased suicide risk (OR = 1.869, p = 0.035), supporting previous findings linking tobacco use to heightened impulsivity, psychiatric comorbidity, and suicidality (12, 14, 63, 64), while prescription drug misuse was similarly associated with elevated risk of suicide attempt (OR = 2.740, p = 0.036).

The main effects of demographic characteristics did not significantly predict suicide attempts in the overall clinical sample. However, consistent with previous literature gender-stratified analyses revealed that women were more affected by certain social determinants (65–67). Specifically, younger age was associated with a reduced likelihood of suicide attempt among women (OR = 0.955, p = 0.007), and having two or more children significantly increased the risk (OR = 2.740, p = 0.036). Additionally, a family history of suicide had a more pronounced effect among women (OR = 2.459, p = 0.028) compared to men, for whom the association was not statistically significant (OR = 1.496, p = 0.672). Notably, gender is not an independent predictor of suicidal behavior, confirming that its influence is mediated by other factors, e.g., depressive disorder or substance abuse.

The distinction between single and multiple suicide attempters further elucidated the cumulative burden of risk factors. Individuals with a history of recurrent suicide attempts exhibited higher rates of depression, bipolar disorder, nicotine dependence, and adverse life events, including a family history of suicide and being born from an unintended pregnancy. Depression emerged as a particularly strong predictor of repeated suicidal behavior (OR = 4.064, p < 0.004) and it reaffirms the central role of depression in chronic suicidality (66, 68–70). Similarly, bipolar disorder (OR = 5.761, p = 0.006) and emotionally unstable personality disorder (OR = 5.132, p = 0.003) were significant predictors of recurrent suicide risk. These findings are consistent with evidence indicating that emotional dysregulation is frequently observed in individuals at heightened risk for suicide (3, 9, 10, 71). The elevated prevalence of emotionally unstable personality disorder among multiple attempters further suggests that impulsivity and affective instability remain clinically salient features in the context of recurrent suicidality (26).

Analysis of suicide motives revealed that relationship conflicts were significantly more frequently cited as reasons for suicide attempts among women compared to men (OR = 2.60, p = 0.035). These findings are consistent with prior research indicating that interpersonal stressors, particularly within intimate relationships, serve as potent emotional triggers for acute suicidal crises (21, 43, 72). Job loss also emerged as a prominent motive, especially among men (OR = 4.074, p = 0.014) and individuals with a history of multiple suicide attempts (OR = 0.438, p = 0.031), suggesting distinct psychosocial risk profiles based on both gender and suicide attempt history (65).

Interestingly, although chronic alcohol abuse was highly prevalent within the sample, it did not significantly predict suicide attempts in the multivariate models (OR = 1.264, p = 0.449). This finding may reflect the role of alcohol as a mediating or exacerbating factor within the context of mood disorders, rather than functioning as an independent predictor of suicidal behavior. Additionally, the high baseline prevalence of alcohol use in psychiatric populations may diminish its discriminatory power in distinguishing between those with and without suicide attempts (29, 73).

### Strengths and limitations

This study offers several important strengths that contribute meaningfully to the existing literature on suicide risk factors within psychiatric populations. First, it adopts a comprehensive biopsychosocial approach, examining a wide range of psychological, social, demographic, familial, and early-life variables, including less frequently studied factors such as being born from an unintended pregnancy, maternal abortion and suicide attempts. The integration of both clinical assessments and self-reported data provides a multidimensional view of suicide risk. Second, the study utilizes a relatively large inpatient sample, allowing for meaningful subgroup comparisons (e.g., single *vs*. multiple attempters, gender-specific patterns). Third, the statistical analysis is robust, including logistic regression models that adjust for multiple covariates, enabling the identification of key predictors such as depression, nicotine dependence, bipolar disorder, and family history of suicide. Importantly, the study also highlights gender differences and recurrent suicide attempts, offering clinically relevant insights for targeted prevention and intervention strategies.

However, several limitations should be considered when interpreting the findings. Most notably, the cross-sectional design limits the ability to establish causal relationships between the identified risk factors and suicide attempts. Longitudinal studies would be required to confirm temporal dynamics and the potential cumulative impact of these factors. Additionally, reliance on self-reported data may introduce recall bias or social desirability bias, particularly in relation to sensitive topics such as substance use, psychiatric history, and early-life experiences. The sample was drawn primarily from psychiatric inpatients, which, while appropriate for exploring high-risk populations, may restrict the generalizability of the results to non-clinical or community samples. Furthermore, cultural and societal factors specific to the Hungarian context—such as stigmatization of mental illness and social attitudes toward abortion and suicide—may influence participant responses and limit the applicability of the findings to other populations.

## Conclusions

Our findings replicate and support previous international (25, 54, 71, 74, 75) and Hungarian studies (3, 4, 9, 11, 72, 73, 76, 77), reinforcing the significance of affective disorders and nicotine dependence in predicting suicide attempts. Previous literature also emphasizes that suicide attempts are frequently associated not only with diagnosable psychiatric disorders as defined by DSM-IV criteria, but also with a high prevalence of subthreshold mental disorders. These subclinical conditions, although not meeting the full diagnostic threshold, warrant serious consideration in the context of suicide prevention strategies. These findings, along with evidence from cited previous studies examining the relationship between smoking and individual-level risk of suicidal behavior, suggest that initiatives aimed at preventing tobacco use and improving the recognition and treatment of depression may contribute to the prevention of suicidality. This study underscores the multifactorial nature of suicidal behavior, integrating psychiatric, sociodemographic, substance-related, and early developmental risk factors. Our research also supports the notion that suicide risk factors interact with one another, and in certain cases, their effects may be synergistic—mutually reinforcing—rather than antagonistic. Similarly, protective factors also appear to amplify each other’s impact, suggesting a cumulative and interactive model of both risk and resilience. Depression emerged as the most robust and consistent predictor of suicide attempts, highlighting the critical importance of early detection and comprehensive treatment of depressive disorders. Nicotine dependence, bipolar disorder, anxiety disorders, and adverse early-life experiences—such as being born from an unintended pregnancy or maternal abortion attempts—also significantly contributed to suicide vulnerability. Socioeconomic stressors, particularly lower educational attainment and financial difficulties were especially relevant among women, while substance use factors were more pronounced among men. Although gender, in itself, was not identified as an independent predictor of suicide attempts—being mediated by other risk factors such as untreated psychiatric disorders—the distinct patterns observed underscore the importance of implementing gender-sensitive prevention strategies. Furthermore, individuals with multiple suicide attempts displayed elevated rates of psychiatric comorbidities and life stressors, emphasizing the need for intensive, long-term post-attempt care. Motivational factors such as interpersonal distress, chronic illness, and existential difficulties were commonly cited, indicating the importance of addressing both psychological symptoms and the psychosocial context of individuals at risk. Despite the study’s limitations, including its cross-sectional design, reliance on self-reported data, and a primarily psychiatric inpatient sample, the findings offer valuable insights into the complex interplay of psychiatric, psychosocial, and developmental contributors to suicide risk. Importantly, this study provides a foundation for practical suicide prevention efforts. Targeted mental health interventions should prioritize depression and include substance use programs—particularly for nicotine cessation—as integral components of care. Addressing socioeconomic disparities through educational and financial support programs is essential, especially for women. Family-based interventions that address intergenerational trauma and a history of suicide within families can play a preventive role, while motivation-specific interventions can provide tailored support for those facing relationship difficulties, chronic illness, or existential distress. Women with a history of abortion also represent a sensitive subgroup requiring specialized counseling and psychological support. Our study also underscores the significance of suicidal behavior among adolescents and youth, emphasizing the need to strengthen prevention efforts within schools and other secondary social settings (e.g., sports clubs). Lastly, robust post-attempt care for individuals with a history of repeated suicide attempts remains a cornerstone of prevention. Future research should expand upon these findings using longitudinal designs, objective clinical assessments, and more culturally diverse samples to enhance the generalizability and impact of suicide prevention strategies. Overall, the study highlights the need for integrated, personalized, and gender-informed interventions that address the diverse and interacting dimensions of suicide risk to reduce the incidence and recurrence of suicidal behavior effectively.

## Data Availability

The raw data supporting the conclusions of this article will be made available by the authors, without undue reservation.
